# Preliminary Immunohistochemical Assessment of Slow-Fiber Distribution and Clustering in Skeletal Muscles of 4-Week- and 6-Month-Old Pigs

**DOI:** 10.3390/ani16182846

**Published:** 2026-09-10

**Authors:** Mutsumi Furukawa, Minami Yanagihara, Miyuko Ohta, Akihiro Kakuzen, Nagomi Takeuchi, Eiki Asai, Ryota Hirakawa, Jahidul Islam, Tomonori Nochi

**Affiliations:** 1 International Education and Research Center for Food and Agricultural Immunology, Graduate School of Agricultural Science, Tohoku University, Sendai 980-8572, Japan; mutsumi.furukawa.c4@tohoku.ac.jp (M.F.); mio0806my@icloud.com (M.Y.); miyuko.ota.t2@dc.tohoku.ac.jp (M.O.); kakuzen.akihiro.p8@dc.tohoku.ac.jp (A.K.); takeuchi.nagomi.s5@dc.tohoku.ac.jp (N.T.); asai.eiki.q3@dc.tohoku.ac.jp (E.A.); ryota.hirakawa.b8@tohoku.ac.jp (R.H.); islam.jahidul.a5@tohoku.ac.jp (J.I.); 2 Laboratory of Animal Functional Morphology, Graduate School of Agricultural Science, Tohoku University, Sendai 980-8572, Japan; 3 Laboratory of Animal Mucosal Immunology, Graduate School of Agricultural Science, Tohoku University, Sendai 980-8572, Japan; 4 Laboratory of Animal Data Science & Innovation, Graduate School of Agricultural Science, Tohoku University, Sendai 980-8572, Japan; 5 Division of Mucosal Vaccines, International Vaccine Design Center, The Institute of Medical Science, The University of Tokyo, Tokyo 108-8639, Japan; 6 Department of Animal Bioscience, University of Guelph, Guelph, ON N1G 2W1, Canada; 7 School of Nutrition and Health Sciences, Taipei Medical University, Taipei City 11031, Taiwan; 8 Tohoku Center for Teaching and Learning, Institute for Excellence in Higher Education, Tohoku University, Sendai 980-8576, Japan

**Keywords:** muscle fiber clustering, skeletal muscles, immunohistochemistry, muscle fibers, myosin, slow-twitch

## Abstract

Type I (slow-twitch) muscle fibers differ in proportion among skeletal muscles, but their local spatial organization remains poorly characterized. This preliminary cross-sectional study investigated whether muscle-specific characteristics were reflected not only in the proportion of slow myosin-positive fibers but also in their clustering architecture. The longissimus, psoas major, semitendinosus, and triceps brachii muscles were collected from pigs at 4 weeks and 6 months of age. All six pigs were female, and live body-weight records were unavailable. Slow myosin-positive muscle fibers (slow fibers) were identified by immunohistochemistry, and adjacent positive fibers were defined as clusters. In pairwise tests at 6 months, the proportion of slow fibers was significantly higher in the psoas major than in the other three muscles, although the main effect of muscle across both ages was not significant (*p* = 0.0565). Slow fibers occurred as either isolated fibers or contiguous clusters in all muscles, but their clustering patterns differed markedly. Notably, the psoas major muscle had a significantly higher proportion of slow fibers in large clusters than the longissimus and semitendinosus muscles at 6 months of age. These findings suggest that muscle-specific characteristics are reflected not only in the proportion of slow fibers but also in their local spatial arrangement. Thus, clustering architecture may provide a complementary histological parameter for characterizing muscle-specific tissue organization in porcine skeletal muscle.

## Simple Summary

The proportion of type I, or slow-twitch, muscle fibers is commonly used to describe the characteristics of skeletal muscles. However, less is known about how these fibers are spatially arranged within muscle tissue. In this preliminary cross-sectional study, four skeletal muscles collected from pigs at 4 weeks and 6 months of age were examined to determine whether the arrangement of slow myosin-positive fibers (slow fibers) differed among muscles and between ages. Slow-twitch fibers were identified by tissue staining and were observed either as isolated fibers or as clusters of neighboring fibers in all four muscles. However, their clustering patterns differed among the muscles. At 6 months of age, the psoas major muscle showed more prominent clustering than the longissimus and semitendinosus muscles. Although only three animals were examined at each age and larger studies are needed to confirm these preliminary findings, the results identify the psoas major as having a distinctive spatial organization of slow fibers and highlight fiber clustering as a potentially informative feature of muscle-specific tissue architecture in pigs.

## 1. Introduction

Skeletal muscle plays essential roles in locomotion, postural maintenance, and energy metabolism, and its contractile and metabolic properties are largely determined by muscle fiber composition [1,2,3]. The proportion of type I (slow-twitch) fibers varies markedly among skeletal muscles and is closely associated with oxidative metabolism, contractile properties, and meat quality [2,3,4,5]. Among these muscles, the psoas major muscle contains a relatively high proportion of type I fibers and exhibits distinct metabolic and meat quality characteristics [6,7,8].

Previous studies have primarily characterized porcine skeletal muscles by quantifying the proportions of type I, type IIA, and type IIX/B fibers and muscle fiber cross-sectional area [1,6,7]. Although these parameters provide valuable quantitative information, they do not describe the spatial organization of muscle fibers. Type I fibers may occur as isolated fibers or contiguous groups, suggesting that their local organization may represent an additional structural feature contributing to muscle-specific characteristics [9]. However, whether the distinctive properties of the psoas major muscle are reflected not only in fiber-type composition but also in local tissue architecture remains unclear. Previous histochemical, immunohistochemical, meat-quality, and transcriptomic studies have examined muscle fiber composition and related characteristics in several porcine skeletal muscles; however, to our knowledge, none have quantified the adjacency-based clustering of slow myosin-positive fibers [1,6,7,8,10,11,12].

Although muscle fiber-type distribution and slow-twitch fiber clusters have been reported in the porcine longissimus muscle, systematic comparisons of type I fiber clustering architecture among different muscles and developmental stages have not been performed [7,8,9]. In the present study, fibers identified as positive for slow myosin by immunohistochemistry are hereafter referred to as slow fibers. We hypothesized that the distinctive characteristics of the psoas major would be reflected in both the proportion and local clustering architecture of these slow fibers.

To test this hypothesis, we compared the psoas major, longissimus, semitendinosus, and triceps brachii muscles at 4 weeks and 6 months of age. Pigs were selected because of their relevance to livestock production and meat quality, and the four muscles were selected to represent different anatomical locations and previously reported fiber-type characteristics. The two ages represented distinct developmental stages: 4 weeks corresponded to the early post-weaning stage, whereas 6 months corresponded to a later growth stage approximately equivalent to the conventional commercial slaughter age. Adjacent slow fibers were defined as clusters, and clustering architecture was quantitatively evaluated using cluster size distribution and the proportion of slow fibers assigned to each cluster-size category. This clustering-based approach complements conventional fiber-type analysis and provides an additional framework for characterizing muscle-specific tissue architecture in porcine skeletal muscle.

## 2. Materials and Methods

### 2.1. Animals and Study Design

All animal experiments were approved by the Animal Care and Use Committee of Tohoku University (approval no. 2022Noudou-020; approved 29 November 2022). Six female Landrace × Large White × Duroc (LWD) crossbred pigs were included in this cross-sectional study: three pigs at 4 weeks of age and three different pigs at 6 months of age. Only female pigs were available for this preliminary study, and sex-related differences were outside the scope of the analysis. All four muscles were collected from each pig, and the individual animal was treated as the biological unit. The two ages represented distinct developmental stages: 4 weeks corresponded to the early post-weaning stage, whereas 6 months corresponded to a later growth stage approximately equivalent to the conventional commercial slaughter age. Body weights were not recorded. The 4-week-old pigs were fed Pig Rush 2; detailed husbandry information, including that for the 6-month-old pigs, was not available. The animals were slaughtered by exsanguination following electrical stunning. Samples were collected from the right-side longissimus, psoas major, semitendinosus, and triceps brachii muscles. The overall experimental and analytical workflow is summarized in Supplementary Figure S1.

### 2.2. Tissue Processing and Histological Staining

Muscle tissues were fixed overnight in 4% paraformaldehyde, dehydrated through a graded ethanol series, cleared in toluene, and embedded in paraffin with muscle fibers oriented in the transverse plane. Paraffin blocks were sectioned at 5 µm using a microtome. After deparaffinization, sections were treated with 0.05% proteinase (Sigma-Aldrich, St. Louis, MO, USA) in Tris-HCl buffer (pH 7.5) at 37 °C for 3 min for antigen retrieval and blocked with 100 mM Tris-HCl (pH 7.4), 150 mM NaCl, and 0.5% blocking reagent (PerkinElmer, Waltham, MA, USA). Sections were incubated overnight at 4 °C with either rabbit anti-laminin antibody (L9393; Sigma-Aldrich, St. Louis, MO, USA; 1:1000) to visualize muscle fiber boundaries or mouse monoclonal anti-myosin skeletal slow antibody (clone NOQ7.5.4D; M8421; Sigma-Aldrich, St. Louis, MO, USA; 1:4000) to detect slow myosin-positive muscle fibers (hereafter referred to as slow fibers). After washing, sections were incubated with Histofine Simple Stain MAX-PO (R) (Nichirei Biosciences, Tokyo, Japan) or MAX-PO (M) reagents (Nichirei Biosciences, Tokyo, Japan). Immunoreactivity was visualized with 3,3′-diaminobenzidine, counterstained with hematoxylin, dehydrated, cleared, mounted with Marinol, and imaged under bright-field conditions using a BZ-9000 microscope (Keyence, Osaka, Japan). Negative-control sections were processed in parallel without the primary antibody.

### 2.3. Quantification of Slow Fibers

The total number of muscle fibers was determined from laminin-stained sections, in which individual fiber boundaries were clearly delineated. Slow fibers were identified in corresponding serial sections immunostained with the anti-skeletal slow myosin antibody. Corresponding regions in the slow myosin- and laminin-stained sections were matched using blood vessels, fascicular boundaries, and section contours as anatomical landmarks. Laminin staining was also used to confirm individual fiber boundaries, particularly when they were unclear in the slow myosin-stained sections. Because muscle fiber size and fascicular architecture, including secondary-fascicle size and the distribution of perimysial connective tissue, differed among muscles and between ages, the objective magnification and imaging area were adjusted to obtain an adequate number of fibers for analysis. Low-magnification images were acquired using a 4× objective, corresponding to a field of view of 3.65 × 2.75 mm. Images used for quantitative analysis were acquired using 10× or 20× objectives, corresponding to fields of view of 1.43 × 1.08 mm and 0.72 × 0.54 mm, respectively.

Four non-overlapping fields were selected systematically at approximately equal intervals across each laminin-stained section before the corresponding slow-myosin-stained sections were examined. The corresponding regions were then identified on the slow-myosin-stained sections using the anatomical landmarks described above; thus, areas with abundant brown staining were not preferentially selected. Muscle fibers and clusters intersecting the image borders were excluded from the analysis. For each muscle sample, the proportion of slow fibers was calculated by dividing the total number of slow fibers by the total number of muscle fibers counted across the four fields. The total and slow fiber counts for each animal and muscle, together with the corresponding means and ranges for each muscle and age group, are provided in Supplementary Table S1.

### 2.4. Analysis of Slow Fiber Clusters

Using laminin staining to identify muscle fiber boundaries, a cluster was defined as either a single slow myosin-positive fiber or a group of adjacent slow fibers sharing a common boundary. Cluster size was defined as the number of slow fibers within each cluster. The mean cluster size for each sample was calculated by dividing the total number of slow fibers by the total number of slow fiber clusters. For each sample, the proportion of slow fibers within each cluster size was calculated. When the number of clusters of size k was defined as $n_{k}$, the proportion of slow fibers in clusters of size k was calculated as $k\times n_{k}$ divided by the total number of slow fibers in all clusters. Clusters were further classified as single-fiber, small, or large clusters. Single-fiber clusters contained one slow fiber, small clusters contained two to four fibers, and large clusters contained five or more slow fibers. This exploratory, data-derived classification was based on inspection of the observed cluster-size distributions: clusters containing up to four slow fibers were consistently observed across muscles and samples, whereas clusters containing five or more slow fibers were observed less consistently.

### 2.5. Statistical Analysis

Statistical analyses were performed using GraphPad Prism version 8.4.3 (GraphPad Software, San Diego, CA, USA). Measurements from multiple fields were aggregated within each muscle sample to obtain a single value for each outcome per animal and muscle. Data were analyzed using a two-way mixed repeated-measures analysis of variance, with muscle as the within-animal factor and age as the between-animal factor, muscle × age as the interaction term, and animal as the subject. Sphericity was not assumed, and the Geisser–Greenhouse correction was applied [13]. Tukey-adjusted comparisons were performed between muscles within each age group, and Sidak-adjusted comparisons were performed between ages within each muscle [14].

## 3. Results

### 3.1. Muscle-Specific Distribution and Proportion of Slow Fibers

**Figure 1:**
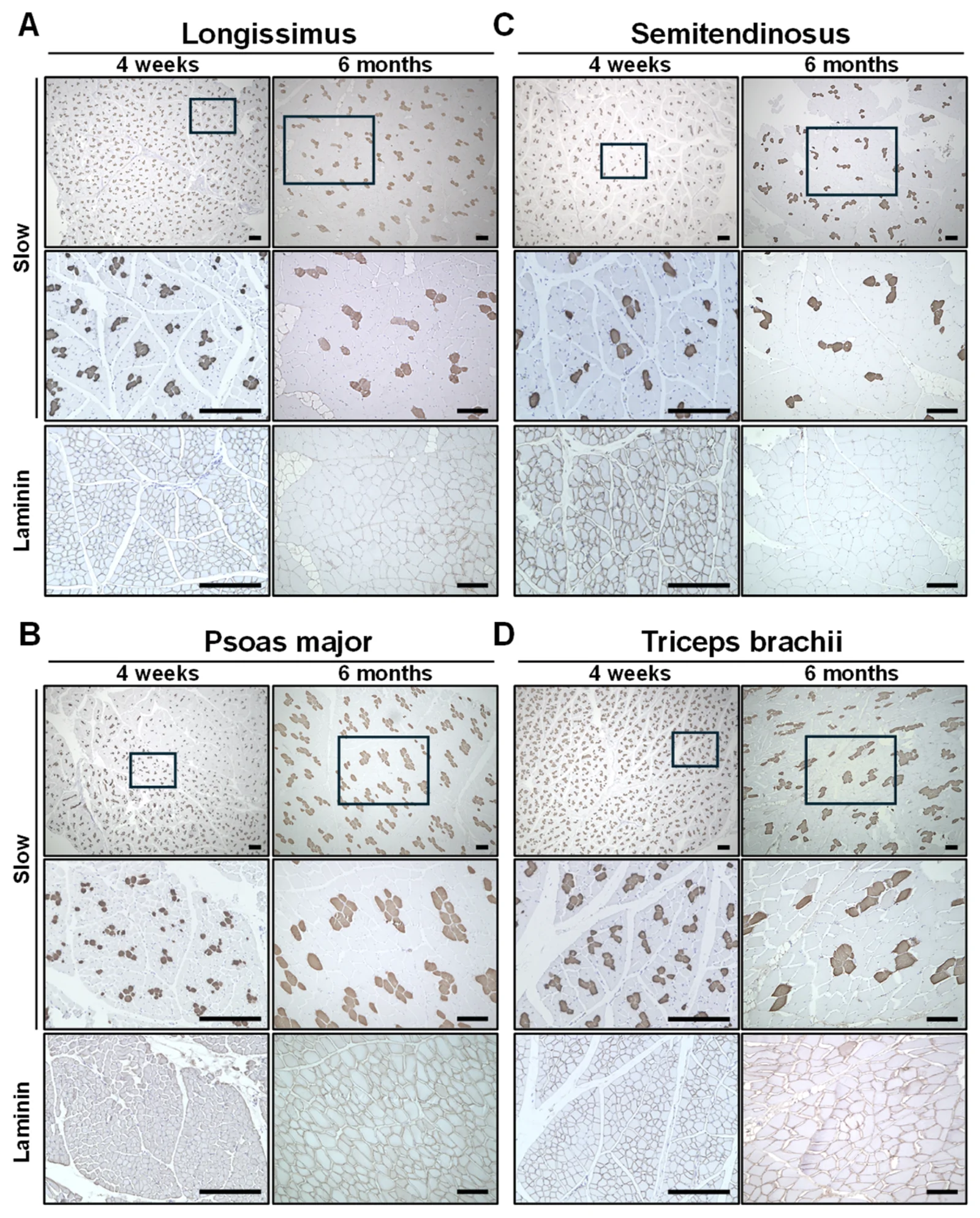
Muscle-specific distribution of slow fibers in 4-week-old and 6-month-old pigs. Representative immunohistochemical images of slow fibers and laminin-stained muscle fiber boundaries in the longissimus (**A**), psoas major (**B**), semitendinosus (**C**), and triceps brachii (**D**) muscles of 4-week-old and 6-month-old pigs. Boxed regions are shown at higher magnification below. Scale bars = 200 μm.

**Figure 2:**
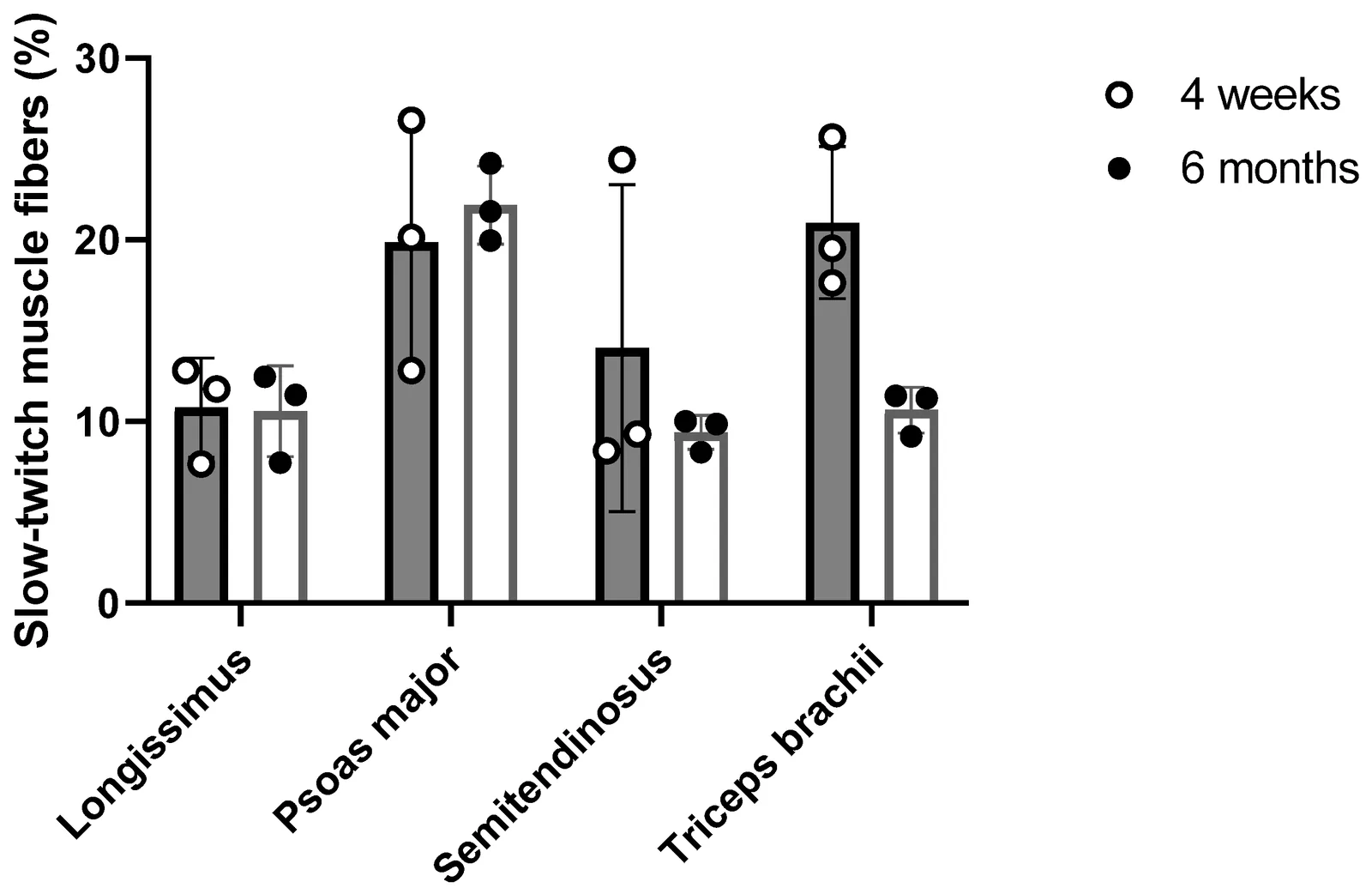
Proportion of slow fibers in the four muscles at 4 weeks and 6 months of age. Open and filled points represent individual 4-week-old and 6-month-old pigs, respectively (*n* = 3 per age group); bars and error bars indicate the mean ± SD. Data were analyzed using a two-way mixed repeated-measures analysis of variance with muscle as the within-animal factor and age as the between-animal factor, followed by Tukey-adjusted comparisons between muscles within each age group. Adjusted *p* values are shown for significant comparisons.

To compare muscle-specific patterns of slow fibers, the longissimus, psoas major, semitendinosus, and triceps brachii muscles were examined (Figure 1A–D). Laminin staining clearly delineated individual muscle fiber boundaries, allowing the proportion of slow fibers to be calculated for each muscle. In both 4-week-old and 6-month-old pigs, slow fibers were observed as either isolated fibers or contiguous clusters. At 4 weeks of age, the proportion of slow fibers was 10.8 ± 2.8% in the longissimus, 14.0 ± 9.0% in the semitendinosus, 19.9 ± 6.8% in the psoas major, and 21.0 ± 4.2% in the triceps brachii muscle. At 6 months of age, the corresponding proportions were 10.6 ± 2.5%, 9.4 ± 0.9%, 22.3 ± 2.8%, and 10.6 ± 1.3%, respectively. No significant muscle-by-age interaction was detected (*p* = 0.2228). The main effect of muscle was not significant (*p* = 0.0565), whereas the main effect of age was significant (*p* = 0.0157). No significant differences among muscles were observed at 4 weeks of age. In pairwise tests at 6 months of age, the psoas major muscle showed a significantly higher proportion of slow fibers than the other muscles examined (adjusted *p* = 0.0251–0.0330; Figure 2). No significant differences between 4 weeks and 6 months of age were detected within any of the four muscles in the Sidak-adjusted pairwise comparisons within any of the four muscles.

### 3.2. Muscle-Specific Differences in Slow Fiber Cluster Architecture

**Figure 3:**
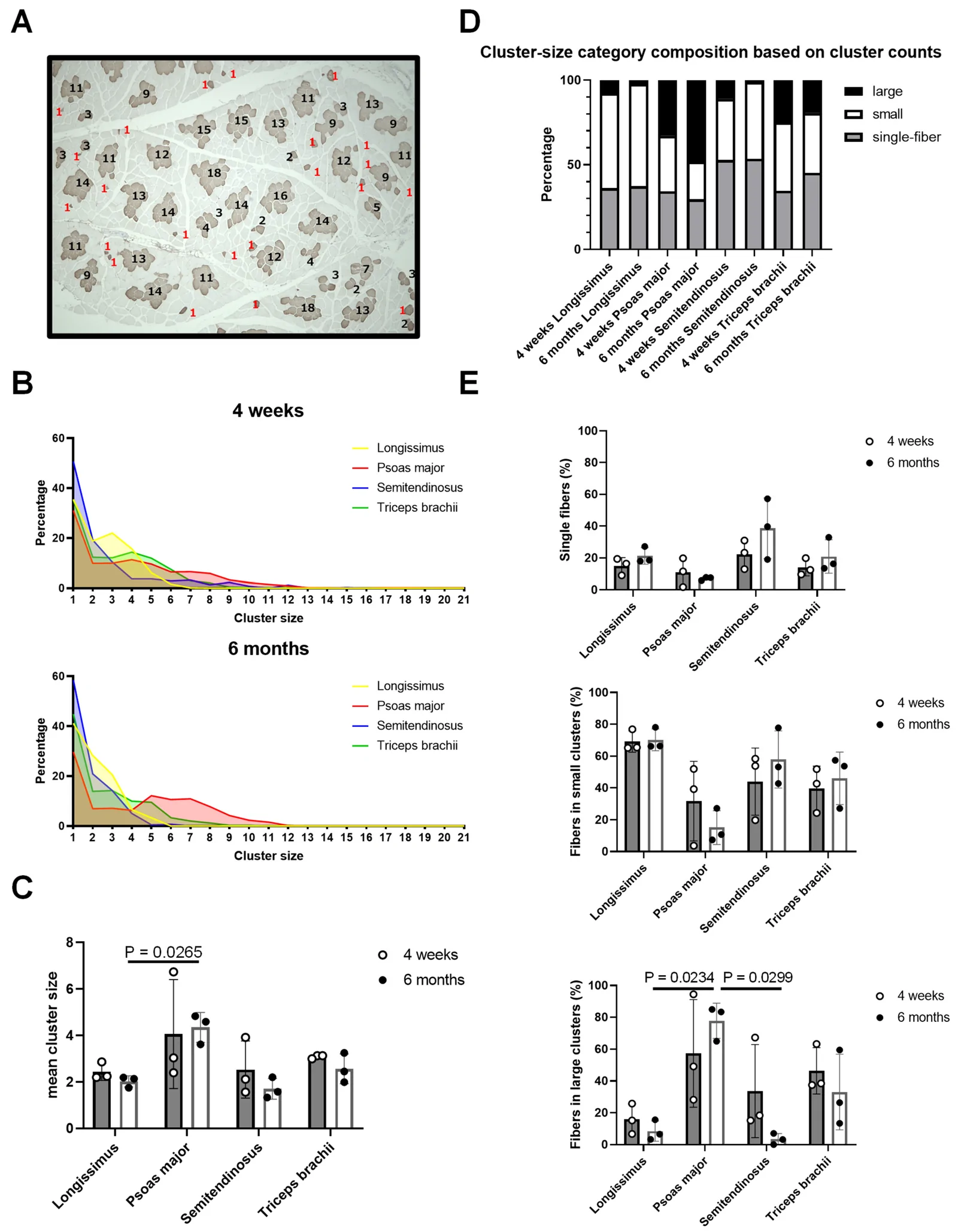
Cluster architecture of slow fibers. (**A**) Representative image of annotated slow fiber clusters in the psoas major muscle of a 6-month-old pig; numbers indicate the number of fibers in each cluster. (**B**) Cluster-size distribution in each muscle at 4 weeks and 6 months of age, shown separately by age. A cluster size of 1 represents a single isolated slow fiber. (**C**) Mean cluster size. (**D**) Cluster-size composition based on cluster counts. Clusters were classified as single-fiber (1 fiber), small (2–4 fibers), or large (≥5 fibers). (**E**) Proportion of slow fibers within each cluster-size category relative to the total number of slow fibers. For clusters containing k fibers, the proportion was calculated as k × n_k_ divided by the total number of slow fibers, where n_k_ represents the number of clusters containing k fibers. Comparisons were performed using a two-way mixed repeated-measures analysis of variance with muscle as the within-animal factor and age as the between-animal factor, followed by Tukey-adjusted comparisons between muscles within each age group. Adjusted *p* values are shown for significant comparisons.

Because slow fibers tended to form clusters, their clustering patterns were evaluated by age and muscle type. Adjacent slow fibers sharing a common boundary were defined as clusters and classified by the number of slow fibers in each cluster (Figure 3A). For each sample, the proportion of each cluster size relative to the total number of clusters was calculated. In the psoas major muscle, clusters containing five to seven slow fibers were present in the 4-week-old pigs and, separately, in the 6-month-old pigs (Figure 3B). In contrast, single-fiber clusters predominated in the semitendinosus muscle at 6 months of age. For mean cluster size, no significant muscle-by-age interaction was detected (*p* = 0.8419), and the main effects of muscle and age were not significant (*p* = 0.0672 and *p* = 0.2572, respectively). In the pairwise comparison at 6 months of age, however, the mean cluster size was significantly greater in the psoas major muscle than in the longissimus muscle (adjusted *p* = 0.0265; Figure 3C). Because clusters containing five or more slow fibers were not consistently detected in all samples, clusters were classified as single-fiber (one slow fiber), small (two to four slow fibers), or large (five or more slow fibers). Large clusters were relatively abundant in the psoas major muscle, whereas most clusters in the longissimus and semitendinosus muscles at 6 months of age were single-fiber or small clusters. The triceps brachii muscle exhibited an intermediate cluster-size distribution. Comparison of cluster categories showed muscle-dependent differences in cluster-size composition (Figure 3D). The proportion of slow fibers within each cluster category relative to the total number of slow fibers in each sample was then calculated (Figure 3E). For the proportion of slow fibers in large clusters, no significant muscle-by-age interaction was detected (*p* = 0.3043), whereas significant main effects of muscle and age were detected (*p* = 0.0144 and *p* = 0.0451, respectively). At 6 months of age, the proportion of slow fibers in large clusters was significantly higher in the psoas major muscle than in the longissimus and semitendinosus muscles (adjusted *p* = 0.0234 and *p* = 0.0299, respectively). Because a higher overall proportion of slow fibers may increase the probability of adjacency and the occurrence of large clusters even under random spatial placement, these findings should not be interpreted as evidence of clustering independent of the overall slow-fiber proportion. No significant differences between 4 weeks and 6 months of age were detected within any muscle in the pairwise tests. These findings indicate that slow fiber clustering architecture differed among muscles at 6 months of age.

## 4. Discussion

The present study showed that muscle-specific differences in slow fibers are reflected not only in their overall proportion but also in their local clustering architecture. Type I (slow-twitch) muscle fibers are generally characterized by oxidative metabolism and fatigue-resistant contractile properties [1,3,10]. Consistent with previous studies, the psoas major muscle exhibited a higher proportion of slow fibers at 6 months of age, consistent with its previously reported oxidative characteristics [7,11,12]. However, the muscle-by-age interactions were not significant, and whether clustering was independent of the slow fiber proportion could not be assessed without a label-randomization analysis [9]. Slow fibers occurred as either isolated fibers or adjacent groups, and their clustering patterns differed among muscles. Moreover, the psoas major muscle contained larger slow fiber clusters, particularly at 6 months of age. These findings suggest that evaluating the local distribution and clustering of slow fibers may provide complementary structural information to conventional analyses of fiber-type composition [9].

Relatively large slow fiber clusters were observed in the psoas major at both developmental stages examined and may represent a muscle-specific structural feature [15,16]. At 6 months of age, the psoas major had a greater mean cluster size than the longissimus and a higher proportion of slow fibers assigned to large clusters than the longissimus and semitendinosus muscles. However, because different animals were examined at the two ages, these cross-sectional observations do not establish that clusters present at 4 weeks were maintained or enlarged during development. Mechanical loading, motor-unit organization, and the local metabolic environment may contribute to the observed clustering pattern, although these factors were not directly evaluated [17,18,19,20]. Moreover, because the longissimus muscle also contributes to stabilization of the vertebral column, postural function cannot be regarded as specific to the psoas major muscle. Although the present study identified muscle-specific differences in slow fiber clustering architecture, their functional significance remains unclear because contractile function and metabolic enzyme activity were not directly evaluated. In addition, slow fibers were identified using a single anti-skeletal slow myosin antibody, and comprehensive myosin heavy-chain-based fiber typing was not performed. The relationship between slow fiber clustering and the spatial distribution of fast-twitch muscle fiber subtypes also remains unclear. Further studies are needed to clarify the mechanisms underlying this muscle-specific clustering pattern.

## 5. Conclusions

In conclusion, this preliminary cross-sectional assessment identified differences in the proportion and local clustering architecture of slow fibers among porcine skeletal muscles. These differences were clearest at 6 months of age, when pairwise comparisons showed that the psoas major muscle had a higher proportion of slow fibers than the other three muscles and a higher proportion of slow fibers in large clusters containing five or more fibers than the longissimus and semitendinosus muscles. Although these preliminary findings require confirmation in studies with larger sample sizes and comprehensive myosin heavy-chain-based fiber typing, they highlight the local spatial organization of slow fibers as a potentially informative histological feature complementary to their overall proportion for characterizing muscle-specific tissue architecture in pigs.

## Data Availability

The aggregated total fiber counts, slow fiber counts, positive-fiber proportions, and cluster metrics for each animal and muscle are provided in Supplementary Table S1.

## Supplementary Materials

The following supporting information can be downloaded at: https://www.mdpi.com/article/10.3390/ani16182846/s1, Figure S1: Experimental design, muscle sampling, histological processing, image acquisition, quantification, and statistical analysis; Table S1: Measurement data for muscle fibers and clusters.
